# Risk of primary liver cancer associated with gallstones and cholecystectomy: A competing risks analysis

**DOI:** 10.1097/MD.0000000000022428

**Published:** 2020-09-25

**Authors:** Tong Liu, Sarah Tan Siyin, Nan Yao, Guoshuai Xu, Yi-Tsun Chen, Ning Duan, Wenqiang Li, Jun Qu, Siqing Liu

**Affiliations:** a Department of General Surgery, Aerospace Center Hospital; b Department of General Surgery, Beijing Children's Hospital, National Center for Children's Health; c Department of Clinic Medicine, Peking University Health Science Center, Beijing; d Department of Hepatobiliary Surgery, Kailuan General Hospital, Tangshan, China.

**Keywords:** cholecystectomy, competing risk models, gallstone disease, incidence, primary liver cancer

## Abstract

Previous research has revealed a positive relationship between GSD, cholecystectomy and primary liver cancer (PLC). However, previous studies had several limitations including the retrospective design, narrow assessment of potential confounders and lack of competing risk models in time-to-event analyses. We conducted a large prospective cohort study to explore the relationship between GSD, cholecystectomy and PLC. A total of 95,021 participants who had not been diagnosed with PLC previously were enrolled from the Kailuan Cohort study. Demographic characteristics and biochemical parameters were recorded at baseline for all participants. We used Cox regression models and competing risk regression models to evaluate the association of GSD and cholecystectomy with the risk PLC. A total of 306 incidental PLC cases were identified during a median follow-up of 9.05 (8.75–9.22) years per participant. Compared with the normal group, the multivariable HRs (95%CI) for the association of GSD and cholecystectomy with PLC were 1.77 (1.05–2.94), 5.25 (1.95–14.17). In the CS model, the multivariable HRs (95%CI) was 1.76 (1.05–2.94) for the association of GSD and cholecystectomy with PLC and 5.25 (1.95–14.17) for GSD and cholecystectomy. Similar results were also obtained in the SD model with corresponding multivariate HRs (95%CI) of 1.75 (1.01–3.00), 5.22 (1.90–14.07) in the GSD group and cholecystectomy group, respectively. GSD and cholecystectomy were associated with an elevated risk of PLC.

Registration number: ChiCTR–TNRC–11001489.

## 1 Introduction

Hepatocellular carcinoma (HCC) and intrahepatic cholangiocarcinoma (ICC) are the most common types of primary liver cancer (PLC) and account for approximately 70% and 15% of PLC cases.^[1]^ Results from Global Burden of Disease Liver Cancer Collaboration revealed that there were 854,000 incidents of liver cancer and 810,000 deaths in 2015 globally.^[2]^ PLC has one of the highest incidences of cancers worldwide and ranks as the fourth leading cause of cancer death in 2015 after lung, colorectal, and stomach cancer. The highest incidences (greater than 20/100,000 persons) of PLC were reported in Asia and Africa, with Chinese registries alone reporting more than half.^[3]^ These areas are suffering from a high prevalence of chronic infection of Hepatitis B Virus (HBV) and Hepatitis C Virus (HCV) as well as an elevated consumption of aflatoxin B_1_ (AFB_1_) contaminated foods. On the contrary, low-rate (less than 10/100,000 persons) PLC areas included Europe and North America, where excessive consumption of alcohol, diabetes and obesity are prevalent.^[3]^

Risk factors including HBV and HCV infection, AFB_1_, alcohol consumption, diabetes and excess body mass index are thought to be key mediators of PLC development.^[4–9]^ However, the etiology of PLC remains largely exclusive apart from the aforementioned factors. Gallstone disease (GSD) as well as cholecystectomy are proven to be closely related to increased risk of several cancers, especially the risk of rectal cancer, colorectal cancer and pancreatic cancer.^[10–14]^ The relationship between GSD, cholecystectomy and PLC is still controversial. Patients with gallstones were observed to have an elevated risk of PLC in recent studies.^[15,16]^ However, Tavani A et al failed to find such a relationship between GSD and PLC.^[17]^ As for cholecystectomy, several studies have demonstrated its strong association with the risk of liver cancer,^[18,19]^ whereas others failed to illustrate a significant association.^[20,13]^ Previous studies had several limitations. First, most were case-control studies which cannot eliminate the possibility of recall bias. Second, the majority of existing studies overestimated the effects of GSD and cholecystectomy on the risk of PLC as other confounders were hardly assessed. Third, during the long period of the follow-up, PLC cases due to the various primary causes of interest are precluded by death due to other causes, thus traditional Cox regression models and Kaplan–Meier survival curves may not appropriately estimate the absolute risks when competing events are censored. No large-scale prospective study has been used a competing risk method to address the question of whether GSD and cholecystectomy elevate the risk of PLC. Therefore, the goal of this study is to examine the relationship between GSD, cholecystectomy and new-onset PLC based on Kailuan study by using competing risk analyses (Trial identification: ChiCTR–TNRC–11001489; Registration number: 11001489).

## 2 Materials and methods

### 2.1 Kailuan study

The Kailuan study is a prospective population-based study in the Kailuan community in Tangshan of China, located 150 km southeast of Beijing. Tangshan is in Hebei Province and has approximately 7.2 million inhabitants. From a socioeconomic perspective, Tangshan can be viewed as a legitimate representation of the Chinese population. The Kailuan Group is a company that is mainly comprised of the coal industry, along with healthcare, education, machine manufacturing and others. This study was designed to explore risk factors related to chronic diseases like hypertension, diabetes and cancers.

### 2.2 Study population

**Figure 1:**
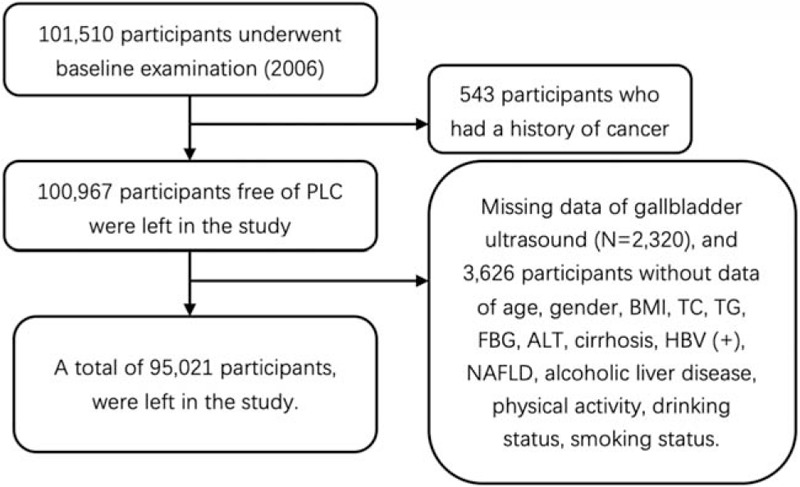
The procedure of participants screening.

Between June 2006 to October 2007, 101,510 participants aged 18 to 98 years underwent a clinical examination and a standardized interview, where relevant data was collected and documented as their baseline. Thereafter, the participants received follow-up examinations and a questionnaire interview biennially. All clinical examinations took place in 11 hospitals that were affiliated with the Kailuan Group. In this current study, 543 participants with a history of cancer at baseline were excluded. Two thousand three hundred twenty participants who lacked gallbladder ultrasound data and 3636 participants without data of potential risk factors for PLC at baseline were also excluded. These potential risk factors include age, gender, body mass index (BMI, in kg/m^2^), total cholesterol (TC, in mmol/L), triglyceride (TG, in mmol/L), fasting blood glucose (FBG, in mmol/L), alanine aminotransferase (ALT, in μ/L), alcoholic liver disease, Hepatitis B virus infection, cirrhosis, nonalcoholic fatty liver disease (NAFLD), physical activity, drinking status, smoking status. A total of 95,021 participants, 75,886 males and 19,135 females, were left in the study. Participants were then divided into 3 groups: normal group, GSD group and cholecystectomy group. This study was approved by the Ethics Review Committee of Kailuan General Hospital as well as Aerospace Center Hospital and was performed according to the Declaration of Helsinki. Written informed consent was obtained from all participants. Details of participant's screening were shown in Figure 1.

### 2.3 Questionnaire assessment

From July 2006 to October 2007, face-to-face interviews were facilitated by trained physicians and research nurses using a standardized questionnaire. Information including age, sex, tobacco consumption, alcohol consumption, physical activity, family medical history (specifically the history of familial cancer) was obtained at baseline. Smoking status was defined as having smoked an average of 1 cigarette/day for more than 1 year. Alcohol consumption was defined as having drunk ≥100 ml/day of ≥50% alcohol content for more than one year. Physical activity was defined as aerobic exercise ≥30 minutes, ≥3 times/week, for more than 1 year. Participants who did not meet the criterion were described as negative for the above classifications.^[21,22]^

### 2.4 Anthropometric measurements and blood pressure measurement

During each interview, the participants height, weight, waist circumference and blood pressure were measured. Height was measured to the nearest 0.1 cm and weight was measured to the nearest 0.1 kg. Body mass index (BMI) was calculated as weight/square of height. Waist circumference was measured to the nearest 0.1 cm midway between the lowest rib and the pelvis during expiration. Two readings of blood pressure were taken at a 5 minutes interval, both on the left arm, with participants in a seated position. The average of the 2 readings was used.

### 2.5 Laboratory assessment

Overnight fasting (≥8 hours) venous blood samples were obtained using vacuum tubes containing EDTA at the baseline examination. Plasma was separated and stored at −80 C for subsequent analyses. The colorimetric enzymatic method (Mind Bioengineering Co Ltd, Shanghai, China) was used to measure the concentrations of both TC and TG. The detectable upper limit was 20.68 and 11.30 mmol/L, respectively. The direct test method (Mind Bioengineering Co. Ltd, Shanghai, China) was used to measure LDL-C and HDL-C concentrations and their detectable upper limit was 12.9 and 3.88 mmol/L, respectively. The hexokinase/glucose-6-phosphate dehydrogenase method was used to measure FBG concentrations. ALT was measured using the enzymatic rate method. For each measurement, the inter-assay coefficient of variation was less than 10%. All of the plasma samples were analyzed at the central laboratory in Kailuan General Hospital using an auto-analyzer (Hitachi 747; Hitachi, Tokyo, Japan).

### 2.6 Metabolic syndrome definition

According to the IDF (International Diabetes Federation) definition, Metabolic syndrome (MetS) is defined as central obesity (waist circumference ≥90 cm in men and ≥80 cm in women) with any 2 of the following 4 factors:

1. Hyperglycemia fasting glucose level >5.6 mmol/L (100 mg/dl) or previously diagnosed diabetes.
2. HDL cholesterol <1.0 mmol/L (40 mg/dl) in men, <1.3 mmol/L (50 mg/dl) in women, or drug treatment for these lipid abnormalities.
3. Blood triglycerides >1.7 mmol/L (150 mg/dl) or drug treatment for elevated triglycerides.
4. Blood pressure >130/85 mm Hg or treatment for previously diagnosed high blood pressure.^[22]^

### 2.7 Type-B ultrasonic examination and assessment

All participants were required to fast for ≥8 hours before the ultrasonic examination. The abdominal region, including liver, gallbladder, pancreas and spleen, were examined by a panel of specialists from Kailuan General Hospital and its 10 affiliated hospitals. Liver cirrhosis, fatty liver, and GSD were diagnosed using real-time ultrasound sonography (PHILIPS HD-15) with 3.5 MHz according to previous clinically established criteria.^[23]^

### 2.8 Outcome ascertainment

During the study period, PLC cases were collected via biennial follow-up examinations of both questionnaires and medical examinations and were classified using ICD-10 codes. Discharge summaries and medical records (obtained from the 11 affiliated hospitals) were evaluated for further information to prevent missed diagnoses. The outcome information of participants who were unable to attend face-to-face follow-ups was obtained from Provincial Vital Statistics Offices (PVSO) which provided death certificates. The diagnosis of incident PLC was checked by reviewing medical archives and pathology reports.^[21,22]^

### 2.9 Statistical analysis

All statistical analysis was performed using a commercially available software program (SAS software, version 9.4). Person-year was calculated from the date of their first examination until the date of incidence (PLC cases), death, or termination of follow-up (December 31, 2018), whichever happened first. Normally distributed variables were presented as means ± standard deviation and compared using one-way analysis of variance (ANOVA). Parameters in the skewed distribution were presented as median ± interquartile range and compared using nonparametric tests. Categorical variables were expressed as percentages and comparison among groups was conducted with χ^2^ test. Multivariate Cox proportional hazard regressions were used to estimate the risk of gallstones and cholecystectomy associated with new-onset PLC. Three models were fitted with adjustments for confounding variables. Model 1 was a univariate analysis. Model 2 was adjusted for age and sex. Model 3 was further adjusted for BMI, FBG, TC, TG, ALT, HBV infection (positive/negative), cirrhosis (yes/no), alcoholic liver disease (yes/no), NAFLD (yes/no), MetS (yes/no), smoking status (yes/no), physical activity (yes/no), and drinking status (yes/no). In epidemiologic data, death can preclude the occurrence of the event of interest and traditional multivariate COX regression model may lead to overestimation of the absolute risk in the presence of competing risks. In these cases, competing risk regression models (cause-specific hazard model and sub-distribution hazard function model), more appropriate to calculate the accurate risk of PLC, were estimated by censoring patients with the competing events(death) and then fitting standard Cox regression models. Statistical tests were 2-sided, and *P* < .05 was recorded as statistically significant.

## 3 Results

### 3.1 Population characteristics

Participant baseline characteristics stratified by gallbladder status (normal, GSD, cholecystectomy) are summarized in Table 1. Compared with the normal group, participants in the GSD group and cholecystectomy group were more likely to be older, to present with higher SBP, WC, BMI, FBG, ALT levels, and lower DBP, LDL-C and TC levels. GSD group and cholecystectomy group were also linked with a higher percentage of cirrhosis, hepatitis B virus infection, physical activity, alcohol drinking, smoking, and NALFD. There was no difference in the HDL-C concentrations and prevalence of the alcoholic liver disease among the 3 groups. It should be noted that the TG concentration and the prevalence of hypertension and MetS were highest in the GSD group.

**Table 1:**
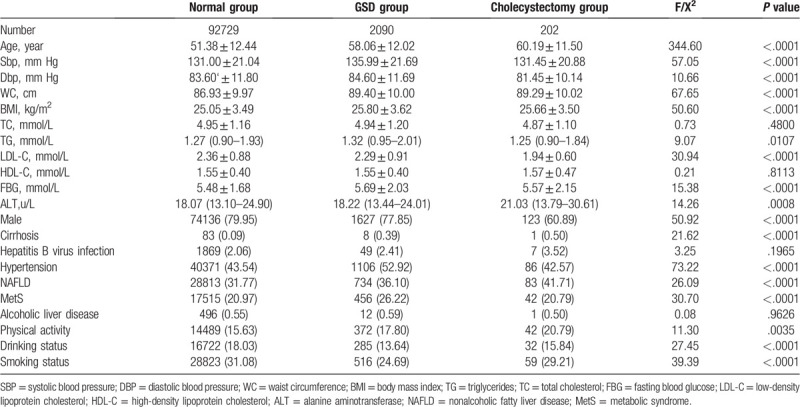
Baseline characteristics of the participants.

### 3.2 Incidence of PLC

During a median follow-up of 9.05(8.75–9.22) years, 306 incidental PLC cases were identified over a total of 828,720 person-years among 95,021 participants. The mean age was 51.55 ± 12.47 with 75,886 (79.86%) males and 19,135 (20.13%) females in our study. The crude incidence of PLC per 10,000 person-years was 3.69 in all participants (1.13/10,000 person-years for females, 4.34/10,000 person-years for males). A clear trend based on gallbladder status was observed, where age- and sex- standardized incidence of PLC increased from 3.48 per 10,000 person-years to 9.43 per 10,000 person-years and 23.41 per 10,000 person-years in the normal group, GSD group and cholecystectomy group, respectively.

### 3.3 Association between gallbladder disease and PLC risk

Table 2 showed the crude and adjusted HRs (95% CI) of new-onset PLC according to gallbladder status using Cox proportional hazards models. In univariate analysis, compared with the normal group, the HRs (95% CI) for the association of GSD and cholecystectomy with PLC were 2.60 (1.57–4.30) and 6.83 (2.54–18.31), respectively. Adjusting for age and sex did not attenuate the trend (Model 2). After the adjustment was made for confounding factors including age, gender, FBG, TC, BMI, TG, ALT, cirrhosis, NAFLD, MetS, HBV infection, alcoholic liver disease, smoking status, drinking status, and physical activity, the association between gallbladder status and PLC was attenuated but remained significant, the corresponding HRs (95%CI) in GSD group and cholecystectomy group were 1.77 (1.05–2.94), 5.25 (1.95–14.17) respectively.

**Table 2:**
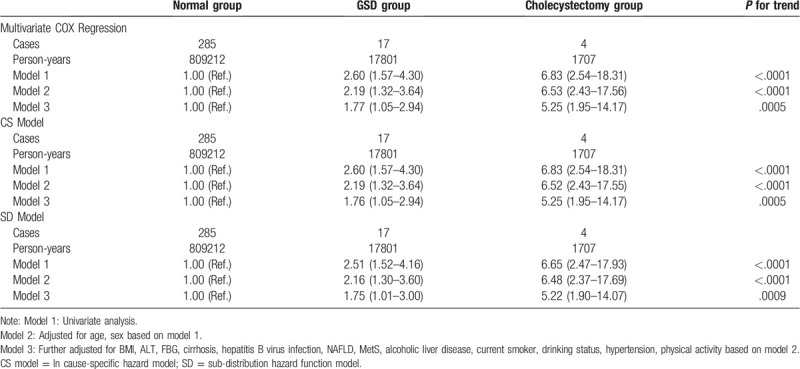
Hazard ratios and 95%confidence interval (CI) for risk of PLC among participants stratified by gallbladder status in different regression models.

### 3.4 The association between gallbladder disease and PLC risk in competing risk regression model

Results of the cause-specific proportional hazard model (CS model) and sub-distribution hazard function model (SD model) for PLC and the competing event were displayed in Table 2. During the median 9.05(8.75–9.22) years of follow-up, 7789 death events were identified before the occurrence of PLC. In the CS model, the HRs (95%CI) for developing PLC among participants in the GSD group and cholecystectomy group were 1.76 (1.05–2.94) and 5.25 (1.95–14.07) in the multivariate-adjusted analysis. In the SD model, after adjustment according to confounding factors as mentioned above, the multivariate HRs (95%CI) for the association of gallbladder status with PLC were 1.75 (1.01–3.00) and 5.22 (1.90–14.07) in the GSD group and cholecystectomy group, respectively.

## 4 Discussion

This study was the first to investigate the relationship between GSD, cholecystectomy, and new-onset PLC among the Chinese population. In this large prospective population-based study, we found that gallstone disease and cholecystectomy were significantly associated with an increased risk of PLC despite being adjusted for suspected confounders including age, gender, FBG, TC, TG, BMI, ALT, NAFLD, MetS, cirrhosis, HBV infection, alcoholic liver disease, smoking status, drinking status, and physical activity. Participants with GSD or who have experienced cholecystectomy had 77% and 425% increased risk of PLC compared with the normal group. The findings of this study were in agreement with the observations made in previous research. A population-based study in US showed that gallstones and cholecystectomy were associated with elevated risk of liver cancer (OR = 2.35 (95% CI: 2.18, 2.54) and OR = 1.26 (95% CI: 1.12, 1.41)). A study conducted in the Swiss showed that people hospitalized with GSD and with post-cholecystectomy status could expect a 117% and 12% increase in the risk of PLC compared with normal participants.^[18]^ However, there is controversy regarding GSD and cholecystectomy being risk factors for PLC. Emily Vogtmann et al reported a statistically significant positive association of GSD with PLC, but failed to find such a relationship between cholecystectomy and PLC.^[24]^ Similarly, a study conducted in Italy and Switzerland by drawing data from a network of case-control studies failed to such a relationship between GSD and PLC (OR = 1.17 (95% CI: 0.83, 1.63).^[17]^ Nonsignificant relationship between cholecystectomy and PLC have also been reported in several studies.^[20,13]^

Competing risks are common in epidemiologic research.^[25]^ In this study, individuals are observed from the time of their baseline examination until the occurrence of PLC, a competing event (death), or time of censoring. Predicting PLC risk grows more difficult when the incidence of competing for events increases, as these competing events might lead to the underestimation of PLC incidence. Thus, when competing events are censored, traditional multivariate COX regression might not appropriately estimate the accurate risk. Competing risk regression models are crucial to estimate the actual individual risk in a time-to-event analysis.^[26,27]^ In this study, both the SD model and the CS model illustrated the circumstances in which the positive association of GSD and cholecystectomy with new-onset PLC cases. There is a substantial difference between the CS model and the SD model, and the application of either model should be dependent on research purposes. CS model should be used when the etiology of a disease is being studied, yet the SD model would be more applicable when the outcome risk of an individual is being predicted.^[28]^

Previous studies have demonstrated the overestimation of standard survival prediction among elderly or frail populations. Application of prognostic models for estimating individual risk requires as much precision as possible to allow for medical decision-making and preventive analytic strategies to be most effective. Competing risk models are encouraged as a standardized tool for the development of predictive models, especially in the elderly population whereby the event of interest may not occur due to a competing event.

GSD is a significant health problem of the biliary tract and is the main reason for inpatient admissions related to gastrointestinal problems. Cholecystectomy is now the gold standard for symptomatic cholelithiasis. The number of surgical procedures for GSD has increased markedly since 1950. The advent of laparoscopic cholecystectomy further drove the cholecystectomy rate in 1989.^[29,30]^ Despite a lack of definitive conclusions for the relationship between GSD, cholecystectomy, and the risk of PLC, previous studies have demonstrated the potential risk of GSD or cholecystectomy with various other cancers including colorectal, esophageal, pancreatic and gastric cancer, resulting in GSD and cholecystectomy being major public health concerns.

Treatment of GSD and PLC is costly and causes much human suffering. Cholecystectomy is almost inevitably performed to cure GSD patients with complications like acute cholecystitis, gallstone ileus, pancreatitis, gallbladder empyema or perforation. To avoid cholecystectomy, effective prevention of GSD has become a prominent aspect of modern medicine. Primary preventions including maintaining an ideal body weight, a regular exercise program, and a low-fat diet have been recommended in previous studies to prevent GSD. With greater lengths taken to prevent GSD, prevalence should decline, thereby also causing incidence of PLC to decrease.

The possible mechanisms for the close association between GSD, cholecystectomy, and PLC are complex and not fully elucidated. Gallstones are known to be closely related to biliary inflammation.^[31]^ A highly significant increase in bile duct diameter and bile duct pressure was demonstrated after cholecystectomy which accounts for subsequent chronic inflammation.^[32]^ Furthermore, the post-cholecystectomy bile duct dilatation increases with time, leading to greater severity of biliary inflammation. Studies have proved that chronic inflammation can fuel the release of reactive oxygen species, chemokines, cytokines, growth factors, and reactive nitrogen intermediates, which are all crucial for tumors development.^[33]^ Besides, it has been speculated that GSD and cholecystectomy lead to the accumulation of bile and secondary bile acids. Studies based on in-vitro or animal experiments have concluded lithocholic acid and deoxycholic acid, with a similar molecular structure as carcinogenic polycyclic aromatic hydrocarbon, can act as carcinogens in the etiology of cancers.^[34,35]^

To our knowledge, this is the first study concerning the relation between GSD, cholecystectomy, and new-onset PLC by using competing risk regression models. The current study is a large-scale prospective cohort study that is less susceptible to selection bias than case-control studies. Other strengths include a broad assessment of potential confounders and high incidence rate of PLC cases, thereby permitting a well-rounded consideration of the related risk factors, as well as the almost 100% follow-up rate during an almost 10-year follow-up period among the target population. This was carried out through the biennial follow-up examinations and extensive healthcare system which included death certificates, medical archives, and health insurance. However, some potential limitations should also be taken note of in this study. First, we could not distinguish between the 2 main different types of primary liver cancers, hepatocellular carcinoma (HCC) and intrahepatic cholangiocarcinoma (ICC), which originates in liver cells and intrahepatic bile duct, respectively. Second, we lacked information about the consumption of AFB_1_ contaminated foods and infection of HCV in baseline examination which may alter the relative risk values of the factors we chose to include. However, previous studies have demonstrated that Chinese patients with HBV are affected 37 times more frequently than Chinese patients with HCV, suggesting that HCV has much less of an effect on the development of PLC in the Chinese population.^[36]^ Third, due to the industrial character of the Kailuan Community, the number of males far exceeded females enrolled in this study. Nevertheless, the influence of imbalance in the results because of sex distribution was minimal as all regression models were adjusted for sex.

In conclusion, this prospective cohort study demonstrated that GSD and cholecystectomy were positively associated with an elevated risk of PLC even when competing risk regression models were applied. Although HBV vaccination was mandatory for all newborns in China beginning in the 1980s, the vaccinated population will not contribute to the current PLC incidence in the population until they reach middle age. Since related risk factors may also pathogenically be connected with the development of the disease, the findings of this study may also be interesting for further elucidating the pathogenesis of PLC and regarded as a clinical screening tool to distinguish individuals with an elevated risk of PLC in the Chinese population.

## Acknowledgments

We thank all staff and participants from the Kailuan study for their vital contributions.

## Author contributions

Tong Liu and Sarah Tan Siyin executed the study and drafted the manuscript. Tong Liu, Sarah Tan Siyin, and Nan Yao participated in the study design and performed the statistical analyses. Guoshuai Xu, Wenqiang Li, Yi-Tsun Chen, and Ning Duan contributed to the discussion. Siqing Liu and Jun Qu reviewed the manuscript.

**Data curation:** Sarah Tan Siyin, Nan Yao, Yi-Tsun Chen, Ning Duan.

**Formal analysis:** Sarah Tan Siyin.

**Investigation:** Wenqiang Li.

**Methodology:** Tong Liu, Yi-Tsun Chen.

**Software:** Tong Liu, Nan Yao, Guoshuai Xu.

**Supervision:** Qu Jun, Siqing Liu.

**Writing – original draft:** Tong Liu, Sarah Tan Siyin.

**Writing – review & editing:** Qu Jun, Siqing Liu.
